# Dental caries in Rwanda: A scoping review

**DOI:** 10.1002/hsr2.1258

**Published:** 2023-05-07

**Authors:** Kehinde K. Kanmodi, Peace Uwambaye, Jimoh Amzat, Afeez A. Salami

**Affiliations:** ^1^ School of Health and Life Sciences Teesside University Middlesbrough UK; ^2^ Faculty of Dentistry University of Puthisastra Phnom Penh Cambodia; ^3^ Cephas Health Research Initiative Inc. Ibadan Nigeria; ^4^ Department of Preventive and Community Dentistry University of Rwanda Kigali Rwanda; ^5^ Department of Sociology Usmanu Danfodiyo University Sokoto Nigeria; ^6^ Department of Sociology University of Johannesburg Johannesburg South Africa; ^7^ Department of Oral and Maxillofacial Surgery University College Hospital Ibadan Nigeria

**Keywords:** Africa, dental caries, dentistry, Rwanda, scoping review, oral health, oral disease, low‐income economy

## Abstract

**Background and Aims:**

Dental caries is an infectious disease affecting virtually all nations, including Rwanda. In Rwanda, the burden of dental caries is an issue of public health concern. To ensure the progressive eradication of the current dental caries burden in Rwanda through an evidence‐based approach, it is imperative to have an overview of the scientific research landscape of dental caries in the country. This study—a scoping review—aims to review the available evidence and gaps on dental caries in Rwanda.

**Methods:**

This scoping review was reported based on the Preferred Reporting Items for systematic reviews and meta‐analyses extension for Scoping Reviews checklist. A systematic search of 11 databases was done to scoop out all literature relevant to the topic. Based on the review's selection criteria, a total of eight peer‐reviewed journal articles were included in the review. The extracted data were collated, summarized, and presented as results.

**Results:**

The analysis of the data extracted from the included articles revealed a high prevalence of dental caries (ranging from 42.42% to 71.5%) in Rwanda. Also, the major pathogens causing dental caries in Rwanda as well as the impact of dental caries on the physical health and quality of life of Rwandans were identified in this review. Furthermore, the reported operative treatment options for dental caries in Rwanda were predominantly nonconservative. Also, no intervention study has been conducted on dental caries in Rwanda.

**Conclusion:**

The findings in this review identify the need for massive public health interventions on dental caries in Rwanda.

## 1 INTRODUCTION

Dental caries is a sugar‐driven, biofilm‐mediated, multifactorial, infectious, transmissible, and preventable disease which is characterized by the phasic destruction of the dental hard tissues.^1^, ^2^, ^3^ Numerous individual risk factors, such as poor oral hygiene practice, susceptible dental morphology (e.g., deep pits and fissures, hypo‐mineralized enamel, etc.), and a poor diet, are significant contributors to the development of caries.^1^, ^2^, ^3^ Dental caries has affected 60%–90% of schoolchildren and almost 100% of adults worldwide.^4^ Also, according to the Global Burden of Disease Study, out of 291 reported diseases and injuries, caries in permanent teeth was found to be the most common oral condition. Untreated caries in deciduous teeth and severe periodontitis in adults came in sixth and tenth place, respectively.^5^ This shows that dental caries is a leading global oral condition.^6^

Untreated caries affects about 3.1 billion people (44% of the world's population) worldwide.^5^ Untreated caries have a significant negative impact on the quality of life of people affected by the disease; also, the management of dental caries is expensive for individuals, families, and society.^6^, ^7^ In areas with a significant socioeconomic gradient, the disease is unevenly distributed.^7^

Dental caries is a condition that develops from a combination of physiological, genetic, environmental, and behavioral factors.^6^ Even though dental caries is generally preventable, its frequency has barely decreased over the past 30 years, which is a severe issue.^6^ In the 2013 national oral health survey in Rwanda, 64.9% of the population had experienced dental caries.^8^ Based on this prevalence rate, it can be asserted that dental caries is a leading disease condition in Rwanda which requires urgent public health attention.

Scientific findings on dental caries are crucial evidence which is vital for the development of strategies, policies, laws, and interventions needed for the control of dental caries disease burden in Rwanda.^9^ Although different studies had been conducted on dental caries in Rwanda; however, no known study had reviewed the available scientific evidence on dental caries in the country, to identify the emerging themes, existing evidence, and evidence gaps concerning the disease. Hence, there is an urgent need for a review of the topic area.

This study aims to do a scoping review of the empirical evidence, emerging themes, and evidence gaps in dental caries research in Rwanda. The findings obtained from this research are very important and fundamental, as they will set the pace for future research on dental caries in Rwanda.

## 2 METHODS

This scoping review adopted the research design developed by Arksey and O'Malley,^10^ and it was documented based on the guidelines of the Preferred Reporting Items for Systematic Reviews and Meta‐Analyses extension for Scoping Reviews checklist.^11^

The scoping review's question was: what are the available empirical evidence and gaps on dental caries in Rwanda?

To answer the scoping review question, an all‐field search of 11 electronic databases was conducted on February 09, 2023, with the aid of “OR” and “AND” Boolean operators, using these search terms: “dental caries,” “tooth decay,” “dental decay,” “caries,” and “Rwanda.” These databases include PubMed, SCOPUS, AMED (The Allied and Complementary Medicine Database), CINAHL Complete, CINAHL Ultimate, APA PsycInfo, APA PsycArticles, Psychology and Behavioral Sciences Collection, SPORTDiscus with Full Text, Dentistry & Oral Sciences Source, and Google Scholar. Supporting Information: Table A1 depicts the search combinations adopted for the database search. From Google Scholar, being a database of gray literature, only the first 100 records were retrieved for the review, as it has been proposed that the first 100 records obtained from a Google Scholar search contain the most relevant set of literature to a search.^12^, ^13^

All retrieved literature was imported into the Rayyan web application for deduplication.^14^ After deduplication, all were screened for relevance and inclusion in the scoping review. Only those literature that were peer‐reviewed journal articles, published in English and with accessible full texts, reporting empirical research findings on dental caries in Rwanda were considered eligible for inclusion into the scoping review. Those literatures that were nonpeer‐reviewed journal articles (e.g., books, book chapters, systematic reviews, letters, opinions, comments, editorials, etc.), those that were published in a non‐English language, those that did not report empirical findings on dental caries in Rwanda, those that reported empirical findings on dental caries outside Rwanda, and those that reported empirical findings on disease conditions that are not dental caries were excluded from the review.

Only those articles that were included in the review were subjected to data extraction. Data concerning the author names, publication year, study design, study objectives, study population attributes (sociodemographic features of the participants), study instruments, study results, and conclusions were obtained using a bespoke data extraction sheet adapted from previous scoping reviews.^15^, ^16^ The extracted data were thereafter collated, summarized, and presented in texts and a table.

## 3 RESULTS

**Figure 1:**
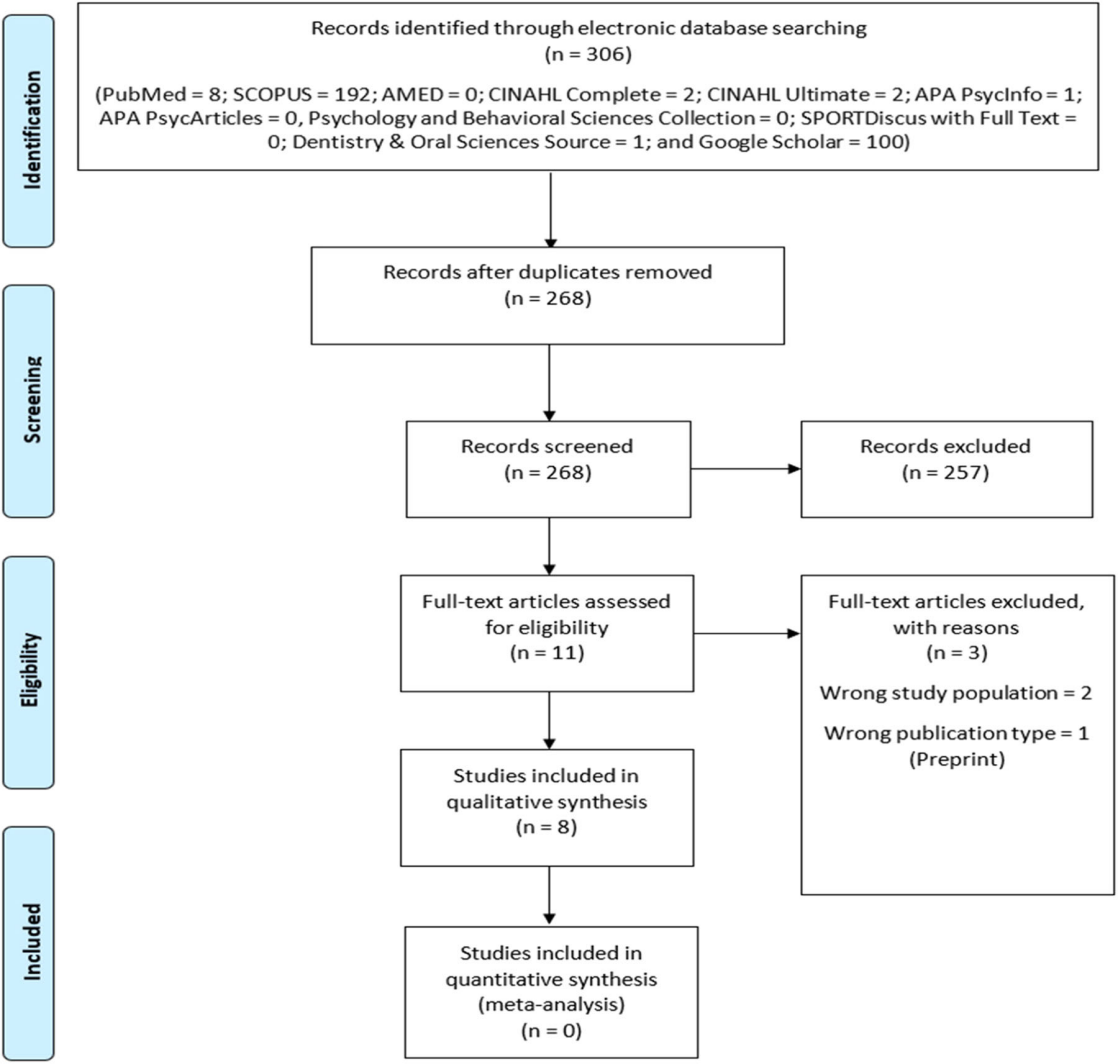
Flow chart diagram.

A total of 306 literatures were retrieved from the database search (PubMed = 8; SCOPUS = 192; AMED = 0; CINAHL Complete = 2; CINAHL Ultimate = 2; APA PsycINFO  = 1; APA PsycArticles = 0, Psychology and Behavioral Sciences Collection = 0; SPORTDiscus with Full Text = 0; Dentistry and Oral Sciences Source = 1; and Google Scholar = 100). A total of 38 duplicates were detected, by the Rayyan software, in the retrieved literature and were deleted. Out of the remaining 268 single‐entry records that were subjected to title and abstract screening, only 11 articles were considered for full‐text screening based on relevance. After the full‐text screening, only 8 peer‐reviewed journal articles were finally included in the review (Supporting Information: Tables A1 and A2). Figure 1 and Table 1 give a summary of the included articles.

**Table 1:** Summary of the articles included in this scoping review.

	Authors and year of publication	Sample size	Country	Objectives	Study design and data source	Study setting	Study population	Conclusion
1	Yadufashije et al.^17^	120	Rwanda	To investigate the association between oral microbial alterations and oral diseases.	Case‐control (primary data)	Hospital‐based	Dental patients, aged 5–46 years, attending Ruhengeri Referral Hospital	Oral microbial alterations contribute to oral diseases.
2	Morgan et al.^8^	2097	Rwanda	To implement the first National Oral Health Survey of Rwanda to assess the oral disease burden and inform oral health promotion strategies.	Cross‐sectional (primary data)	Community‐based	The study population consisted of 2097 participants (61.1% female), aged 2–40+ years, representing all provinces in Rwanda and the capital city Kigali	The international effort contributed to building oral health research capacity resulted in a national oral health database of oral disease burden in Rwanda. This information is essential for developing oral disease prevention and management strategies as well as oral health workforce and infrastructure.
3	Mukashyaka et al.^18^	287	Rwanda	To describe the management of patients seeking care for dental caries at Butaro District Hospital (BDH) in rural Rwanda	Cross‐sectional (primary data)	Hospital‐based	Patients (age range: ≤5 years to >21 years) presenting with a diagnosis of dental caries at BDH during the period of study	Caries prevention and care should be prioritized through a developed community program on oral health. We recommend introducing advanced training, equipment, and materials for dental caries management other than tooth extraction, and increasing the number of qualified dentists
4	Hackley et al.^4^	2097	Rwanda	National Oral Health Survey of Rwanda (NOHSR) data were investigated for associations of socio‐demographic characteristics, personal oral hygiene practices, oral health outcomes, and oral health quality of life indicators	Cross‐sectional (secondary data)	Community‐based	The study population consisted of 2097 participants (61.1% female), aged 2–40+ years, representing all provinces in Rwanda and the capital city Kigali	Socioeconomic, individual, and workforce characteristics are important considerations when assessing oral health outcomes. This study investigated social demographic disparities in relation to oral health‐related behaviors and outcomes. This information can help guide oral health care programming in Rwanda.
5	Uwayezu et al.^19^	226	Rwanda	To determine the prevalence of dental caries and associated risk factors among children with disabilities	Cross sectional (primary data)	Nongovernmental organization‐based	The study population was all children aged between 7 and 20 years old living with physical disabilities; learning, intellectual and developmental disabilities; deafness, blindness, and hearing impairment disabilities both males and females of NYANZA HVP GATAGARA	The findings show that dental caries are reality and prevalent among children living with disabilities in Rwanda.
6	Hitimana and Ndayisenga^20^	281	Rwanda	To determine dental caries prevalence and associated risk factors among adult outpatients who attended Gakoma district hospital in Gisagara district of Rwanda from November 2016 to January 2017	Cross‐sectional (primary data)	Hospital‐based	Adult outpatients (age not specified) who attended Gakoma District Hospital in Gisagara District of Rwanda from November 2016 to January 2017	The prevalence of dental caries was found to be high in the study population and the multifactorial risk factors associated with its development were found to be poor oral hygiene, frequent consumption of sugary snacks and beverages, gender, and old age
7	Uwayezu et al.^21^	660	Rwanda	To assess the prevalence of dental caries, its associated factors, and treatment needs among school‐aged children at Kimironko 2 primary school	Cross‐sectional (secondary data)	School‐based	School‐aged children (age range: ≤6 years to ≥13 years) at Kimironko 2 primary school	Dental caries is prevalent among children of Kimironko II Primary School
8	Yadufashije et al.^22^	68	Rwanda	To identify sugar fermenting bacteria in the oral cavity and their antibiotic susceptibility pattern, assess the association with sugar fermenter bacteria and dental caries and evaluate dental caries outcomes among children	Cross‐sectional (primary data)	Hospital‐based	Children, aged 4–10 years, with or without oral diseases at Ruhengeri referral hospital	Sugar consumption favors the growth of sugar fermenter bacteria that cause dental caries among children. Dental caries is associated with adverse oral health outcomes among children.

### 3.1 Sources of the included studies

All the included studies were authored by researchers affiliated to Rwanda institutions. Three studies were coauthored by researchers from Kenyan institutions,^17^, ^20^, ^22^ one study was coauthored by a researcher from a Moroccoan institution,^17^ three studies were coauthored by researchers from institutions in the United States Hackley et al.^4^, ^8^, ^18^ and one study was coauthored by a researcher from Indian institution.^20^

### 3.2 Design of the included studies

All the included studies adopted a cross‐sectional study design, except the study by Yadufashije et al.^17^ which adopted a case‐control study design. All, except two studies,^4^, ^21^ were based on primary data. Also, four studies were hospital‐based,^17^, ^18^, ^19^, ^20^, ^22^ two were community‐based,^4^, ^8^ one was school‐based,^21^ and one was conducted in a nongovernmental organization (NGO).^19^

### 3.3 Populations studied in Rwanda

Overall, the studies investigated a total of 3139 people in Rwanda. Only two studies investigated child populations only,^21^, ^22^ only one study investigated adult population only,^20^ and only five studies investigated a mixed population of children and adults.^4^, ^8^, ^17^, ^18^, ^19^

Also, four studies investigated dental patients,^17^, ^18^, ^20^, ^22^ two studies investigated households (i.e., community people),^4^, ^8^ one study investigated school children,^21^ and one study investigated people (predominantly children) living with disability under the care of an NGO.^19^

### 3.4 Prevalence of dental caries in Rwanda

Most of the selected articles studied the prevalence of dental caries and associated risk factors. Only six studies reported the prevalence of dental caries in Rwanda. Two studies reported the prevalence rates of dental caries in children—the study by Uwayezu et al.,^21^ which reported a rate of 42.42% among apparently school children not living with disability, and the study by Uwayezu et al.,^19^ which reported a rate of 42.4% among people (predominantly children) living with disability.

Only one study, by Hitimana and Ndayisenga,^20^ reported a dental caries prevalence (71.5%) among adults.

Only three studies reported the prevalence rates of dental caries among mixed‐population groups. Among these population groups, the study by Hackley et al.^4^ reported untreated caries prevalence rate of 54.3%, the study by Morgan et al.^8^ reported dental caries experience prevalence rate of 64.9%, and the study by Yadufashije et al.^17^ reported dental caries (unspecified type) prevalence rate of 56.6%.

### 3.5 Major pathogens causing dental caries in Rwanda

Only one study, by Yadufashije et al.,^22^ investigated the major bacterial pathogens causing dental caries among the Rwandan population. In their study, they reported five sugar fermenter bacteria as the statistically significant (*p* < 0.05) cause of dental caries among Rwandan children. These bacteria were *Staphylococcus aureus*, *Streptococcus mutans*, *Enterobacter aerogenes*, *Serratia marcescens*, and *Klebsiella pneumoniae*.

### 3.6 Determinants of dental caries in Rwanda

Only four studies investigated the determinants of dental caries in Rwanda.^4^, ^19^, ^20^, ^21^ In these studies, age,^4^, ^20^ personal oral hygiene practices,^4^, ^20^, ^21^ frequency of sugary diet intake,^19^, ^20^ gender,^20^, ^21^ oral health status,^19^ and dental check‐up status^21^ were the statistically significant determinants (*p* < 0.05) that were identified.

### 3.7 Impact of dental caries on Rwandan population

Only three studies investigated the impact of dental caries on Rwandan population.^4^, ^8^, ^22^ The study by Yadufashije et al.^22^ reported tooth pain, tooth loss, tooth color change, infection, speaking regression, regression in academic performance, and dentinal hypersensitivity among children affected with dental caries. The other two studies^4^, ^8^ reported the impact of dental caries on the quality of life of people with dental caries—these studies reported that dental caries experience was associated with: embarrassment; pain; difficulty in chewing and speaking; difficulty in attending school; and difficulty in participating in social activities among those affected with the condition.

### 3.8 Management of dental caries in Rwanda

Only one study, by Mukashyaka et al.,^18^ reported the management of dental caries among a Rwandan population group. In the study, oral hygiene counseling, dental extraction, dental filling, dental cleaning, drug (antibiotic and analgesic) prescription, and/or referral to other health facilities were the treatment modalities delivered to the patients. Furthermore, denture delivery, root canal treatment, and dental crowning were not indicated among the treatment modalities.

## 4 DISCUSSION

This scoping review indicated a high prevalence of dental caries in Rwanda due to high‐risk behavior concerning dental caries. The prevalence of dental caries reported was >41% rate among the studied populations. This finding is similar to other studies in Africa concerning the prevalence of dental caries.^23^, ^24^, ^25^, ^26^, ^27^ In Sudan, a prevalence of dental caries of slightly over 50% among preschool children has been reported.^24^ In a meta‐analysis of studies conducted on dental caries prevalence in Ethiopia, a comparatively high pooled prevalence (>40%) of dental caries was recorded.^27^ Similarly, in a meta‐analysis of dental caries prevalence in East Africa (including Eritrea, Sudan and Tanzania, Uganda), a pooled dental caries prevalence of 45.7% was recorded in the subregion.^26^

Another systematic review and meta‐synthesis of 30 studies, by Kimmie‐Dhansay and Bhayat,^25^ revealed discrepancies in dental caries prevalence but a relatively high incidence among 12‐year‐olds in Africa. The meta‐synthesis reported a high prevalence in urban centers compared to rural areas, and a higher prevalence in studies conducted after 2015. The significant submission is that the burden of dental caries is increasing in Africa. However, global oral health inequalities indicate it is less prevalent in Africa compared to other continents.^28^ Nevertheless, Africa carries a high rate of untreated caries due to low health infrastructure and limited access to essential oral healthcare services.^29^ A study elsewhere (in the United States) showed that oral healthcare is a significant symbol of social inequality.^30^ The report indicated that most people with relatively low socioeconomic status, uninsured, and minority ethnic groups have relatively low access to quality oral health care. Incidentally, people living in the lower rung of society disproportionately share common risk factors of dental caries such as high sugar consumption and poor oral hygiene.

This review also collated other dental caries determinants (apart from sugary diet intake), which include personal oral hygiene practices, gender, oral health status, and dental check‐up status. Health determinants are contextual issues which explain the propensity to risk factors operating at the individual, community and global levels.^31^, ^32^ Elamin et al.^33^ revealed that increased age, low maternal education, low parental involvement, low overall socioeconomic status, low frequency of tooth brushing, poor oral habits, and sugar consumption were common determinants of dental caries (see also^34^, ^35^).

This review revealed the common bacteria causing dental caries among Rwandan populations. In general, some oral microbes are in constant flux but serve as causative agents of dental caries.^36^ Dental caries is a microbe‐mediated oral disease. Chen et al.^37^ explained a four‐factor theory of oral microorganisms, oral environment, host, and time responsible for dental caries. In general, sweet food consumption—a major risk factor for dental caries—was significantly associated with a decayed, missing, and filled tooth (DMFT) index.^38^, ^39^

This review also documents the pathological impact of dental caries on Rwandan populations, such as tooth pain, tooth loss, and tooth color change, among others. Dental caries also impacts the quality of life and induces negative social experiences. Ballo et al.^34^ noted that dental caries significantly predicts oral health‐related quality of life (OHRQoL) in preschool children. The impact is mostly negative, especially at the advanced stages of dental caries. However, the patient's age and household income are associated with the impact on quality of life.^40^ Kastenbom et al.^41^ showed significantly high economic costs, apart from problems relating to depression among the caries active group. Bukhari^42^ also noted physiological limitations and psychological discomfort as significant impacts. Painful aching has been the most common physical discomfort. The significant impacts are many including pain, physical discomfort, psychological concerns, treatment, and social costs.

Last, this review documented the management of dental caries including dental hygiene counseling, dental extraction, dental filling, dental cleaning, and drug (antibiotic and analgesic) prescription. However, more conservative treatment options like root canal treatment and crowning were not provided in any of the reviewed studies from Rwanda. While noting the high prevalence of dental caries, with high levels of tooth loss, this review suggests a case of limited access to preventive oral healthcare in Rwanda. Hence, there is a need for massive preventive and restorative oral healthcare programs in Rwanda. The other preventive strategies include a community‐based approach based on oral health education and atraumatic restorative treatments in primary health care (PHC) centers.^43^

### 4.1 Limitations of the review

This scoping review has its limitations. First, only eight articles met the inclusion criteria for this review despite the high prevalence of dental caries in Rwanda. This indicates a low volume of research on dental caries in Rwanda; hence, limiting the robustness of this review. However, due to the scientific rigor of the review process, coupled with the multidisciplinary expertise of the authors (Kehinde K. Kanmodi is a general dental practitioner and public health researcher; Peace Uwambaye is a community dentist; Jimoh Amzat is a medical sociologist; and Afeez A. Salami is an oral and maxillofacial surgery registrar) which were channeled into the review process, a very robust body of evidence was synthesized from the sparse literature. Second, gray literatures were not included into this review; hence, limiting the robustness of the review. However, the exclusion of gray literatures was deliberate as the authors sought to review empirical and peer‐reviewed scientific evidence only, to boost the credibility of the findings obtained in this review.^44^

### 4.2 Conclusion

Like many other African countries, the prevalence of dental caries is high in Rwanda. This study also collated determinants of dental caries including sugar consumption, personal oral hygiene practices, gender, and oral health status. This review also documents the pathological impact of dental caries on Rwandan population including tooth pain, tooth loss, and tooth color change, among others. Dental caries also impact the quality of life and induce negative social experiences. The significant impacts are many including pain, physical discomfort, psychological concerns, and negative social experience. There is a need to improve preventive and restorative oral healthcare programs using a community‐based approach which incorporates oral health education and atraumatic restorative treatments in primary healthcare. The programmatic interventions should also consider the social determinants of oral health to mitigate the negative influence of social variables (maternal education and age) and hygienic practice (improved toothbrushing).

## AUTHOR CONTRIBUTIONS

**Kehinde K. Kanmodi**: Conceptualization; data curation; formal analysis; funding acquisition; investigation; methodology; project administration; resources; software; supervision; validation; visualization; writing—original draft; writing—review and editing. **Peace Uwambaye**: Conceptualization; formal analysis; investigation; methodology; resources; writing—original draft. **Jimoh Amzat**: Resources; writing—original draft; writing—review and editing. **Afeez A. Salami**: Data curation; investigation; methodology; resources; software.

## CONFLICTS OF INTEREST STATEMENT

Kehinde K. Kanmodi is an Editorial Board member of Health Science Reports and a coauthor of this article. To minimize bias, they were excluded from all editorial decision‐making related to the acceptance of this article for publication. The other authors declare no conflict of interest.

## TRANSPARENCY STATEMENT

The lead author Kehinde Kazeem Kanmodi affirms that this manuscript is an honest, accurate, and transparent account of the study being reported; that no important aspects of the study have been omitted; and that any discrepancies from the study as planned (and, if relevant, registered) have been explained.

## Supporting information

Supporting information.

## Data Availability

Data sharing is not applicable to this article as no new data were created or analyzed in this study.
