# Supplementary material for: Dental caries in Rwanda: A scoping review
Source: Health Sci Rep. 2023 May 7;6(5):e1258. doi: 10.1002/hsr2.1258 (PMC10164754; doi:10.1002/hsr2.1258)
Supplement: Supplementary file 1 — Supporting information. [file HSR2-6-e1258-s001.docx]

**APPENDIX**

**Table A1. Search strings for database search**

| **PubMed** | | | |
| --- | --- | --- | --- |
| **Tag** | **Subject search** | **Search Field** | **Search String** |
| #1 | Dental caries | All fields | (((Dental caries) OR (tooth decay)) OR (dental decay)) OR (caries) |
| #2 | Rwanda | All fields | (Rwanda) |
| #3 | #1 AND #2 |  | (#1) AND (#2) |
| **SCOPUS** | | | |
| #1 | Dental caries | All fields | ( ALL ( dental AND caries ) OR ALL ( tooth AND decay ) OR ALL ( dental AND decay ) OR ALL ( caries ) ) |
| #2 | Rwanda | All fields | ALL ( Rwanda ) |
| #3 | #1 AND #2 |  | (#1) AND (#2) |
| **AMED (The Allied and Complementary Medicine Database) [via EBSCO*Host* Interface]** | | | |
| S1 | Dental caries | All fields | Dental caries OR tooth decay OR dental decay OR caries |
| S2 | Rwanda | All fields | Rwanda |
| S3 | S1 AND S2 |  | S1 AND S2 |
| **CINAHL Complete [via EBSCO*Host* Interface]** | | | |
| S1 | Dental caries | All fields | Dental caries OR tooth decay OR dental decay OR caries |
| S2 | Rwanda | All fields | Rwanda |
| S3 | S1 AND S2 |  | S1 AND S2 |
| **CINAHL Ultimate [via EBSCO*Host* Interface]** | | | |
| S1 | Dental caries | All fields | Dental caries OR tooth decay OR dental decay OR caries |
| S2 | Rwanda | All fields | Rwanda |
| S3 | S1 AND S2 |  | S1 AND S2 |
| **APA PsycInfo [via EBSCO*Host* Interface]** | | | |
| S1 | Dental caries | All fields | Dental caries OR tooth decay OR dental decay OR caries |
| S2 | Rwanda | All fields | Rwanda |
| S3 | S1 AND S2 |  | S1 AND S2 |
| **APA PsycArticles [via EBSCO*Host* Interface]** | | | |
| S1 | Dental caries | All fields | Dental caries OR tooth decay OR dental decay OR caries |
| S2 | Rwanda | All fields | Rwanda |
| S3 | S1 AND S2 |  | S1 AND S2 |
| **Psychology and Behavioral Sciences Collection [via EBSCO*Host* Interface]** | | | |
| S1 | Dental caries | All fields | Dental caries OR tooth decay OR dental decay OR caries |
| S2 | Rwanda | All fields | Rwanda |
| S3 | S1 AND S2 |  | S1 AND S2 |
| **SPORTDiscus with Full Text [via EBSCO*Host* Interface]** | | | |
| S1 | Dental caries | All fields | Dental caries OR tooth decay OR dental decay OR caries |
| S2 | Rwanda | All fields | Rwanda |
| S3 | S1 AND S2 |  | S1 AND S2 |
| **Dentistry & Oral Sciences Source [via EBSCO*Host* Interface]** | | | |
| S1 | Dental caries | All fields | Dental caries OR tooth decay OR dental decay OR caries |
| S2 | Rwanda | All fields | Rwanda |
| S3 | S1 AND S2 |  | S1 AND S2 |
| **Google Scholar** | | | |
| S1 | Dental caries | All fields | Dental caries OR tooth decay OR dental decay OR caries |
| S2 | Rwanda | All fields | Rwanda |
| S3 | S1 AND S2 |  | (Dental caries OR tooth decay OR dental decay OR caries) AND (Rwanda) |

**Table A2. Publications included for full text screening and their screening outcome.**

| **Item** | **Citations** | **Screening Outcome** | |
| --- | --- | --- | --- |
|  |  | **Include** | **Exclude** |
| 1 | Uwayezu, D., Gatarayiha, A., & Nzayirambaho, M. (2020). Prevalence of dental caries and associated risk factors in children living with disabilities in Rwanda: a cross-sectional study. *The Pan African medical journal*, *36*, 193. https://doi.org/10.11604/pamj.2020.36.193.24166 | Yes |  |
| 2 | Morgan, J. P., Isyagi, M., Ntaganira, J., Gatarayiha, A., Pagni, S. E., Roomian, T. C., Finkelman, M., Steffensen, J. E. M., Barrow, J. R., Mumena, C. H., & Hackley, D. M. (2018). Building oral health research infrastructure: the first national oral health survey of Rwanda. *Global health action*, *11*(1), 1477249. https://doi.org/10.1080/16549716.2018.1477249 | Yes |  |
| 3 | Mukashyaka, C., Uzabakiriho, B., Amoroso, C. L., Mpunga, T., Odhiambo, J., Mukashema, P., Seymour, B. A., Sindayigaya, J.deD., & Hedt-Gauthier, B. L. (2015). Dental caries management at a rural district hospital in northern Rwanda: a neglected disease. *Public health action*, *5*(3), 158–161. https://doi.org/10.5588/pha.15.0022 | Yes |  |
| 4 | Yadufashije, C., Uwase, D., Muhimpundu, L., Izere, C., Mucumbitsi, J., Munyeshyaka, E., Twagirumukiza, G., Mwanzia, L. N., Migabo, H., & Mala, A. O. (2022). Profiles of sugar fermenting bacteria of the oral cavity among children with dental caries attending stomatology services at Ruhengeri referral hospital in Musanze District, Northern Rwanda. *The Nigerian postgraduate medical journal*, *29*(3), 236–243. https://doi.org/10.4103/npmj.npmj_78_22 | Yes |  |
| 5 | Hackley, D. M., Jain, S., Pagni, S. E., Finkelman, M., Ntaganira, J., & Morgan, J. P. (2021). Oral health conditions and correlates: a National Oral Health Survey of Rwanda. *Global health action*, *14*(1), 1904628. https://doi.org/10.1080/16549716.2021.1904628 | Yes |  |
| 6 | Hitimana, E., & Ndayisenga, L. (2022). Prevalence of dental caries and associated risk factors among adult outpatients attending Gakoma district hospital, Rwanda. *Journal of Orofacial Research*, 38-43. | Yes |  |
| 7 | Bikoroti, J. B., Byimana, J., Ndatinya, A., Bayisenga, J., Rwibasira, J. M., Gasangwa, A., ... & Tuyizere, E. (2017). Dental and periodontal diseases among United Nations personnel in mission: a fifteen months experience of Rwanda Level 2 Hospital Bria in Central African Republic. *International Journal of Dental Medicine*, *3*(2), 4-10. |  | Yes (Wrong study population) |
| 8 | Yadufashije, C., Mucumbitsi, J., Uwimana, M. J. U., Muhimpundu, L., Mwanzia, L. N., Ndayambaje, M., ... & Mala, A. O. (2022). Association with oral microbial alteration and oral disease among patients attending Ruhengeri referral hospital, Rwanda: A case–control study. *Biomedical and Biotechnology Research Journal (BBRJ)*, *6*(1), 126. | Yes |  |
| 9 | Goodson, J. M., Shi, P., Mumena, C. H., Haq, A., & Razzaque, M. S. (2017). Dietary phosphorus burden increases cariogenesis independent of vitamin D uptake. *The Journal of steroid biochemistry and molecular biology*, *167*, 33–38. https://doi.org/10.1016/j.jsbmb.2016.10.006 |  | Yes (Wrong study population) |
| 10 | Uwayezu, D., Uwambaye, P., Uwitonze, A. M., Murererehe, J., Nzabonimana, E., Ineza, M. C., ... & Gatarayiha, A. (2021). Prevalence of Dental Caries, its Associated Risk Factors and Treatment Needs among School Aged Children at Kimironko II Primary School, Kigali, Rwanda. *Rwanda Journal of Medicine and Health Sciences*, *4*(3), 341-346. | Yes |  |
| 11 | Murererehe, J., Kolisa, Y. M., Niragire, F., & Yengopal, V. (2022). Prevalence of dental caries and associated risk factors among HIV-positive and HIV-negative adults at an HIV clinic in Kigali, Rwanda. *medRxiv*, 2022-10. |  | Yes (Wrong publication type) |
